# The Flywheel Effect of Gender Role Expectations in Diverse Work Groups

**DOI:** 10.3389/fpsyg.2019.00976

**Published:** 2019-05-07

**Authors:** Hans van Dijk, Marloes L. van Engen

**Affiliations:** ^1^ Department of Organization Studies, Tilburg University, Tilburg, Netherlands; ^2^ Department of Human Resource Studies, Tilburg University, Tilburg, Netherlands

**Keywords:** gender role expectations, impression formation motivation, team performance, diverse teams, stereotypes

## Abstract

Popular press suggests that gender diversity benefits the performance of work groups. However, decades of research indicate that such performance benefits of gender diversity are anything but a given. To account for this incongruity, in this conceptual paper we argue that the performance of gender-diverse work groups is often inhibited by self-reinforcing gender role expectations. We use the analogy of a flywheel to illustrate how gender role expectations tend to reinforce themselves *via* three mechanisms. Specifically, we argue that gender role expectations shape (1) the allocation of jobs, tasks, and responsibilities, (2) the behavior of perceivers, and (3) the behavior of target women and men. In turn, these three consequences of gender role expectations tend to confirm the initial gender role expectations, thus creating an automatic, self-reinforcing flywheel effect. Such self-reinforcing gender role expectations provide superficial impressions of individual women’s and men’s actual knowledge and abilities at best. We therefore further propose that each of the three mechanisms of the flywheel of gender role expectations negatively affects group performance to the extent that gender role expectations inaccurately capture group members’ actual knowledge and abilities. Because the extent to which work group members rely on gender role expectations depends on how they form impressions of others, we propose that individuals’ motivation to form accurate impressions is crucial for inhibiting the flywheel of gender role expectations. We close by advancing an agenda for future research on each of the three areas of interest in our conceptual analysis: the flywheel effect of gender role expectations, the consequences of this flywheel effect for group functioning, and ways to motivate group members to form accurate impressions.

Although popular press proclaims that gender diversity benefits the performance of work groups (e.g., teams, departments, and organizations; see Catalyst, 2004), these statements seem based more on wishes than reality (Eagly, 2016). A meta-analysis of 56 studies that in total represent 7,141 gender-diverse teams (the most proximal unit to assess the consequences of gender diversity) showed a non-significant relationship between gender diversity and team performance (*r* = −0.01; van Dijk et al., 2012). There are, however, a number of plausible arguments why gender diversity should benefit work group performance.

First, in most organizations, individuals are selected based on knowledge and abilities. As gender is often not indicative of individual performance, the optimal work group composition should be a mix with the women and men highest in knowledge and abilities (cf. Lindberg et al., 2010). An underrepresentation of women or men in a certain work group, hence, often reflects a certain amount of “false positive error” (selecting a candidate that is not the best for the job) and “false negative error” (not selecting the best candidate for the job) in selection.

Second, given that men and women tend to be socialized differently (Eagly, 1987), they are likely to hold different knowledge, perspectives, and ideas (cf. May et al., 2018). If gender-diverse work groups are able to pool and use the corresponding richness and variety in information, they should be able to make better decisions than gender-homogeneous work groups (cf. van Knippenberg et al., 2004).

Third, most work groups target male as well as female clients (i.e., customers, consumers). In harboring men as well as women, gender-diverse work groups should be better able to understand and cater to the needs of their clients (cf. Ely and Thomas, 2001).

The lack of support for positive effects of gender diversity on work group performance therefore begs the question why the potential of gender diversity is not realized. In this article, we address this question and offer a way forward for researchers and practitioners to better understand what is needed for unlocking the potential performance benefits of gender diversity in work groups.

Specifically, we contend that the main obstacle for the performance of gender-diverse work groups is the self-reinforcing nature of gender role expectations. Ample research in the past decades has shown that gender stereotypes create role expectations in workplaces regarding the behavior of men and women on tasks and positions (Heilman; 1983; Eagly, 1987; Ridgeway, 1991; Eagly and Karau, 2002; Biernat et al., 2010). We argue that these role expectations reinforce themselves by behaving like a flywheel (i.e., a heavy wheel that keeps rotating with little effort after it has gained momentum, e.g., a potter’s wheel): *via* a series of bigger and smaller pushes, momentum is created and attained, such that gender role expectations (1) operate autonomously and (2) sustain and reinforce themselves.

We identify three mechanisms *via* which gender role expectations tend to reinforce themselves in gender-diverse work groups. The first is the influence of gender role expectations in the *allocation of jobs, tasks, and responsibilities* (cf. social role theory, Eagly, 1987; role congruity theory, Eagly and Karau, 2002; status construction theory, Ridgeway, 1991); the second is the influence of gender role expectations in *the behavior of perceivers* (cf. expectation states theory, Berger et al., 1974; stereotype content model, Fiske et al., 2002; backlash, Rudman et al., 2012); and the third is the influence of gender role expectations in *the behavior of women and men* (cf. stereotype threat, Hoyt and Murphy, 2016; fear of backlash, Akinola et al., 2018).

Because each mechanism is grounded in generalized impressions of the knowledge and abilities of women and men based on their gender, the mechanisms are always affected by a certain degree of inaccuracy regarding the actual knowledge and abilities of target women and men. Higher degrees of inaccuracy are likely to exacerbate the extent to which jobs, tasks, and responsibilities are allocated to less-knowledgeable group members, and the extent to which the behaviors of perceivers and of women and men disrupt performance. As a consequence, we propose that gender role expectations harm work group performance to the extent that gender role expectations inaccurately capture target women and men’s knowledge and abilities. To decrease the likelihood that perceives let their gender role expectations influence their impressions of women’s and men’s knowledge and abilities and form more accurate impressions of women’s and men’s knowledge and abilities, we argue that it is crucial that perceivers are motivated to form accurate impressions of each other.

Our conceptual analysis provides three main contributions to the literature. First, whereas gender role expectations are known to negatively affect the position and performance of women and men in stereotype-incongruent roles, we extend these insights by applying them to gender-diverse work groups and argue that gender role expectations in gender-diverse work groups operate like a flywheel. Second, by building theory on this flywheel effect of gender role expectations in gender-diverse work groups, we assert that it is the inaccuracy of gender role expectations that cause gender-diverse work groups to fail in realizing their full potential. Third, in building theory and setting a future research agenda on how to inhibit or alter self-reinforcing gender role expectations, we provide theoretically as well as practically novel suggestions for how to improve the functioning of gender-diverse work groups.

## The Flywheel of Gender Role Expectations

Research on the performance of (gender-)diverse work groups has commonly adopted a bi-theoretical approach to explain why and how gender diversity may positively or negatively affect group performance (van Knippenberg et al., 2004; van Knippenberg and Schippers, 2007). The information/decision-making perspective suggests that diverse work groups hold a richer variety in knowledge and information. When members are able to pool and combine the variety in knowledge and information, diverse work groups should be able to make better decisions and hence outperform homogeneous work groups. By contrast, the social categorization perspective suggests that differences between group members increase the likelihood that group members perceive each other as different, which can lead to the emergence of subgroups, and subsequently increase subgroup conflicts and decrease cohesion as well as the pooling and integration of knowledge and information.

Although this bi-theoretical approach enables accounting for positive as well as negative outcomes, it has omitted how stereotypes and corresponding role expectations shape behaviors, dynamics, and outcomes of diverse work groups (van Dijk et al., 2017). Role expectations represent societally crafted associations and beliefs that enable perceivers to navigate through a world of infinite complexity based on people’s characteristics. As such, gender role expectations help perceivers reduce complexity by making inferences about women and men regarding their attitudes, behaviors, skills, etc. based on their gender (Eagly, 1987; Eagly et al., 2000; Haines et al., 2016). By focusing on a person’s gender to form an impression of a target person, gender role expectations reduce the amount of time and effort that they would otherwise need to spend on individuation (van Dijk et al., 2017). In work groups, gender role expectations can therefore benefit perceivers by inferring female and male group members’ knowledge and abilities, and using that to determine whom to ask for advice and whose input to ignore (cf. van Dijk et al., 2018).

However, forming impressions based on gender role expectations also comes at a cost. Although gender stereotypes tend to be accurate in predicting overall differences between women and men at the societal level (Jussim et al., 2015), at the individual level, stereotype-based impressions are at best superficial generalizations and at worst sexist and highly inaccurate. For example, whereas men overall may be more assertive compared to women, one cannot assume that all male members of a gender-diverse work group are more assertive than all female group members. Despite these potential costs, perceivers do tend to rely on gender role expectations in forming impressions of individual women and men because gender role expectations consume few cognitive resources, and because individuating information is not always available. Insight into how gender role expectations shape group behavior and dynamics is therefore crucial for understanding how gender diversity shapes work group performance.

Many consequences of gender role expectations are well understood and documented in the form of meta-analyses, reviews, and books (e.g., Eagly et al., 2000; Wood and Eagly, 2012). However, studies that focus on the organizational context mainly look at the consequences of gender role expectations for individuals (e.g., obtaining a leadership position, e.g., Eagly and Karau, 2002; individual performance, e.g., Chatman et al., 2008) and stay relatively mute to the role of gender role expectations in processes and outcomes at the work group level (van Dijk et al., 2017).

Furthermore, studies that focus on the consequences of gender role expectations tend to adopt a static approach by assessing how gender role expectations shape certain behaviors and outcomes related to gender inequality. Although there is an occasional reference to potential vicious cycles or downward spirals (e.g., Martell et al., 1996), such dynamic relationships remain under-theorized and are insufficiently explored.

In this conceptual contribution, we argue that the self-reinforcing nature of gender role expectations demands more attention, since it provides insight into why gender role expectations are so pervasive and may cause so many gender-diverse groups to fail reaching their potential. We use the analogy of a flywheel to explain the self-reinforcing nature of gender role expectations. The heavier a flywheel, the more effort is needed to make it spin, but also the harder it is to slow it down once it rotates. Once a flywheel has gained momentum, the flywheel only requires an occasional reinforcement to keep rotating. A flywheel effect thus refers to the continuation of rotations even after the original stimulus has been removed, such that the flywheel (1) operates autonomously and (2) reinforces itself (cf. Collins, 2001). It is because of these two aspects that we deem this a more appropriate and fitting analogy to illustrate how gender role expectations tend to reinforce themselves compared to the hollower terms of vicious cycles and downward spirals. Specifically, we assert that these two aspects of a flywheel capture the tendency of gender role expectations to (1) automatically (i.e., sub-consciously) evoke decisions, behaviors, and interactions that, in turn, (2) confirm and thereby reinforce the very same gender role expectations.

**Figure 1:**
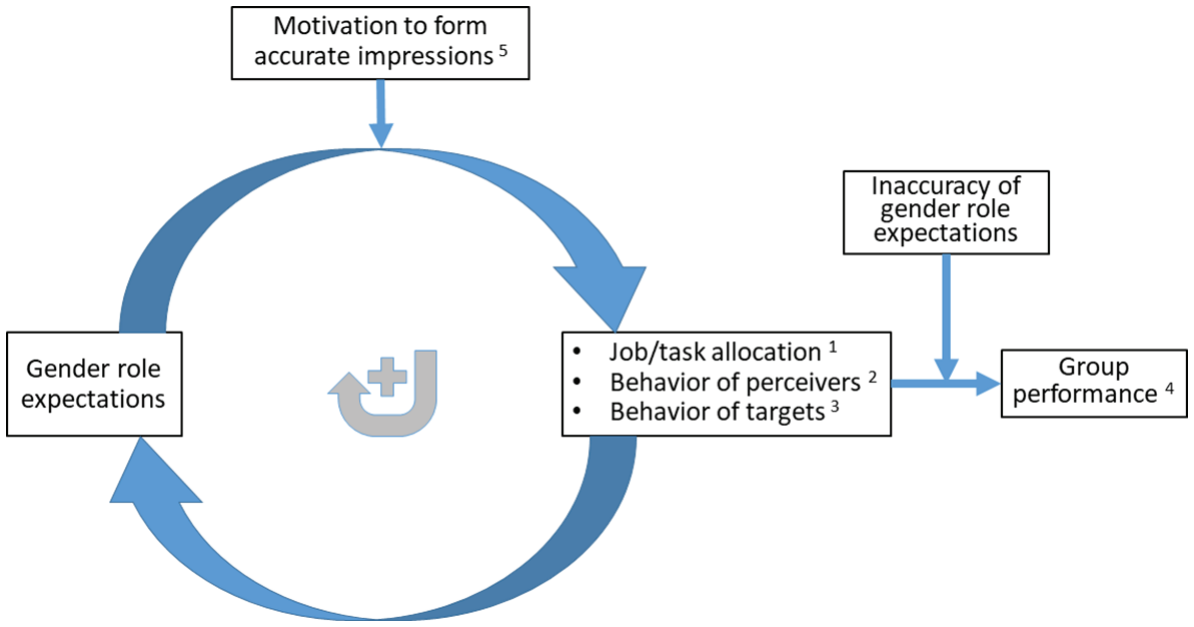
The flywheel of gender role expectations, group performance, and impression formation motivation. *Note*: numbers indicate the corresponding propositions.

Figure 1 shows our conceptual model. In the following, we first discuss the self-reinforcing nature of gender role expectations, and subsequently discuss how the flywheel of gender role expectations shapes group performance.

## Ways in which Gender Role Expectations are Self-Reinforcing

We propose that there are three mechanisms *via* which gender role expectations tend to behave like a flywheel by reinforcing themselves in gender-diverse groups. These mechanisms are as follows: (1) the allocation of jobs, tasks, and responsibilities, (2) the behavior of perceivers, and (3) the behavior of target women and men. As is recommended for building theory in order to understand a phenomenon (Sparrowe and Mayer, 2011), we base our arguments on different theories that shed a light on the self-reinforcing nature of gender role expectations from a different angle.

### The Allocation of Jobs, Tasks, and Responsibilities

Each group and each organization usually aims to recruit the best (i.e., most knowledgeable, skilled, able) person for a job or task, and likewise allocate responsibilities based on people’s competencies and expertise. However, in a focus on finding the best person, there is a caveat, because a perceiver’s judgment and evaluation of a target person to a large extent tends to be based on the perceiver’s own bias and beliefs (Scullen et al., 2000). Gender role expectations form a prominent source of such biases and beliefs. For example, meta-analytical evidence shows that men are preferred over equally able women for male-typed jobs (but not for female-typed or integrated jobs) (Koch et al., 2015). These findings are in line with the lack-of-fit model (Heilman, 1983) and the role congruity theory (Eagly and Karau, 2002), both of which indicate that men are more likely to be recruited and selected for, or promoted into, a leadership position because the male role fits better or is more congruent with the leadership role in the eyes of perceivers.

Ironically, it is the subsequent underrepresentation of women in leadership positions that maintains and reinforces the gender role expectations that men are more suitable for leadership positions, if only because women are not granted the opportunity to prove their worth. Indeed, social role theory (Eagly, 1987) as well as status construction theory (Ridgeway, 1991) suggest that the mere observation of men dominating leadership positions and women being overrepresented in supportive (e.g., administration) or nurturing (e.g., caretaker) roles created, reinforced, and continues to uphold the belief or expectation that men are more suited for agentic and leadership roles and that women fit better in supportive and nurturing roles.

Such a flywheel effect of gender role expectations is not only likely to occur in the allocation of positions but also in many other allocation and decision-making processes in organizations. Consider, for example, performance evaluations (e.g., Lyness and Heilman, 2006; Bosquet et al., 2018), reward allocations (e.g., Castilla, 2008; Abraham, 2017), and promotion decisions (e.g., Roth et al., 2012). It is no coincidence that such evaluations and decisions also tend to be affected by gender role expectations, given that higher performance evaluations are likely to yield higher reward allocations, more chances on a promotion, as well as more chances on being allocated a prominent job, task, or responsibility. Gender role expectations can thus shape the allocation of jobs, tasks, and responsibilities by affecting performance evaluations in an earlier stage that, over time, may be crucial in determining who gets the job.

When looking at the effects of gender role expectations on the allocation of jobs, tasks, and responsibilities in a static way (i.e., at a fixed point in time), such effects may appear small or even nonexistent. However, because of the self-reinforcing nature of allocation and decision-making processes, the resulting cumulative effect over time may very well explain why the proportion of women tends to be lower the more one ascends the hierarchical ladder in organizations (Martell et al., 1996; Agars, 2004; Ridgeway, 2011).

In sum, we propose that gender role expectations shape decisions regarding the allocation of jobs, tasks, and responsibilities, such that gender role expectations tend to maintain and reinforce themselves. Men are more likely to be selected for jobs, tasks, and responsibilities that are congruent with the male gender role, whereas women are more likely to be selected for jobs, tasks, and responsibilities congruent with the female gender role. In subsequently observing the gender-confirming allocation of men and women, gender role beliefs and expectations are likely to be sustained and reinforced. The following flywheel effect is thereby created:

Proposition 1: Gender role expectations tend to reinforce themselves via the allocation of jobs, tasks, and/or responsibilities: women and men are less likely to be appointed to a job, task, and/or responsibility that are incongruent with their gender role, and the consequent underrepresentation of persons in gender-incongruent roles maintains and reinforces gender role expectations.

### The Behavior of Perceivers

Our first proposition suggests that it can already be difficult for women and men to obtain a job, task, or position that does not correspond with gender role expectations. But if women and men do obtain such a gender role-incongruent position, we argue that there is a second, complementary mechanism in the flywheel that makes it difficult for them to sustain such a position. This mechanism consists of a collection of behaviors of perceivers that tend to confirm and reinforce gender role expectations.

Specifically, expectation states theory (Berger et al., 1974) suggests that gender role expectations cause perceivers to display supportive or more critical behavior toward a person, depending on the extent to which gender role expectations suggest that the person holds task-relevant knowledge and abilities. The more these gender role expectations suggest that a target person has the knowledge and abilities for a task (e.g., men on male-typed tasks), the more the perceiver will support the person by granting the person opportunities to act, evaluating the person more positively, and being more influenced by the person (Correll and Ridgeway, 2003; Wittenbaum and Bowman, 2005; cf. Cuddy et al., 2007). If, however, gender role expectations suggest that a person does not hold task-relevant knowledge and abilities (e.g., men on female-typed tasks), such a person tends to be victim of various unsupportive behaviors of perceivers. Perceivers may, for example, ignore or interrupt the person, evaluate her or him more negatively, and/or discredit the person (Foschi, 2000). Women and men in gender role-incongruent positions thus are more likely to be the recipients of unsupportive behaviors by perceivers. In turn, such unsupportive behaviors make it more likely that women and men in gender role-incongruent positions fail or quit.

Furthermore, research in backlash suggests that unsupportive behaviors toward people in gender-incongruent positions are not only grounded in gender-based inferences of knowledge and abilities in relation to the task context, but also in more general gender role beliefs. Backlash refers to social and economic reprisals for behaving counter-stereotypically, which can range from the unsupportive behaviors mentioned earlier to discrimination and sabotage (Rudman and Phelan, 2008). Meta-analytical evidence showed that women who explicitly display dominance in male-typed task contexts (i.e., where the majority of workers tend to be men) tend to experience backlash (Williams and Tiedens, 2016). Other research suggests that especially women in high-status male-typed task contexts are likely to suffer from backlash, because their counter-stereotypical presence in such task contexts threatens men’s high-status position in society (Rudman et al., 2012). Based on a series of experiments, Rudman and colleagues concluded that “defending the gender hierarchy is a primary motive for backlash” and that, for example, “prejudice against female leaders stems from perceived status violations” (p. 175). There is less research on backlash for men in counter-stereotypical roles, but in line with the argument that backlash is motivated by a defense of the gender hierarchy, those studies overall show that men experience backlash when displaying communal behavior in female-typed task contexts (Moss-Racusin, 2015).

Taken together, expectation states theory and research in backlash suggest that women and men in gender role-incongruent positions are more likely to be subject to unsupportive behaviors from perceivers compared to women and men in gender role-congruent positions. Such unsupportive behaviors increase the chance that women and men in gender role-incongruent positions fail and/or drop out of their position. Moreover, women and men in gender role-incongruent positions tend to be penalized for displaying behavior that is required for the job or task because it is gender role-incongruent, and therefore become subject of more unsupportive behaviors. We therefore propose that perceivers tend to be supportive toward women and men in gender role-congruent positions, which enables such women and men to do well and remain in their position. By contrast, perceivers tend to be unsupportive of women and men in gender role-incongruent positions, which makes it more likely that such women and men fail and drop out of their positions. The successes of women and men in gender role-congruent positions and the failures of women and men in gender role-incongruent positions, in turn, confirm and reinforce the initial gender role expectations. The behavior of perceivers thus contributes to a flywheel effect that maintains and reinforces gender role expectations:

Proposition 2: Gender role expectations tend to reinforce themselves via the behavior of perceivers: perceivers tend to be less supportive toward women and men in gender role-incongruent positions compared to women and men in gender role-congruent positions, which makes women and men in gender role-incongruent positions more likely to fail and thus maintains and reinforces the gender role expectations.

### The Behavior of Individuals

Because individuals are exposed to gender role expectations from their cradle onward, they are often unaware of them and may frequently display behaviors that confirm gender role expectations. For example, women [men] may have been raised to be more modest [assertive] and submissive [dominant], and in showing such behavior reinforce gender role expectations. Social role theory (Wood and Eagly, 2012) suggests that men and women have also internalized gender role expectations and therefore may even prefer to display gender role-confirming behavior.

Even if persons have achieved a gender role-incongruent position, they often remain affected by gender role expectations. The aim of backlash against women and men in gender role-incongruent positions who display counter-stereotypical behavior is to make them behave according to gender norms. Many studies show that the mere fear of backlash already tends to cause women and men to adjust their behavior, up to the point where they may display gender conformity (Rudman and Fairchild, 2004). For example, studies have shown that a fear of backlash caused women to avoid behaving assertively in negotiations on behalf of themselves (Amanatullah and Morris, 2010), limit power displays in political and organizational settings (Brescoll, 2011), distance themselves from supporting subordinate women (Derks et al., 2016), and delegate less compared to men, which hampered performance (Akinola et al., 2018).

Another reason why women and men may display gender role-confirming behavior is stereotype threat. Stereotype threat refers to “the psychological experience of a person who, while engaged in a task, is aware of a stereotype about his or her identity group suggesting that he or she will not perform well in that task” (Roberson and Kulik, 2007, p. 26). Research on stereotype threat (Steele and Aronson, 1995) suggests that aiming to disprove stereotypes can paradoxically also lead to their confirmation. Specifically, several meta-analyses (Wheeler and Petty, 2001; Walton and Spencer, 2009) indicate that stereotype threat negatively affects women and men’s performance on more complex gender role-incongruent tasks. There are different explanations for why stereotype threat hampers the performance of women and men on such gender role-incongruent tasks. One explanation suggests that gender role expectations create an awareness among women and men in gender role-incongruent positions that they are expected to perform less well compared to women and men in gender role-congruent positions. This awareness is experienced as a threat that taxes the working memory of women and men in gender role-incongruent positions, and thereby inhibits their ability to perform well (Schmader et al., 2008). Another, potentially complementary explanation is that the awareness of gender role expectations has a demotivating effect. Being demotivated may not just hamper performance, but can even cause women and men to disengage and/or avoid gender role-incongruent positions (Hoyt and Murphy, 2016).

Regardless of whether target women and men tend to display stereotype-confirming behavior because they have been socialized that way, because they fear backlash, or because of stereotype threat, in each case the outcome is that a target person’s own behavior is reinforced to be congruent with gender role expectations. In turn, such gender role-congruent behaviors maintain and reinforce the initial gender role expectations, thus contributing to the flywheel effect of gender role expectations. We therefore propose:

Proposition 3: Gender role expectations tend to reinforce themselves via the behavior of individuals: gender role expectations tend to cause women and men in gender role-incongruent positions to display gender role-congruent behavior, which maintains and reinforces the gender role expectations.

## The Consequences of the Flywheel of Gender Role Expectations for Gender-Diverse Groups

Although studies on the consequences of gender role expectations tend to focus almost exclusively on how gender role expectations affect (outcomes of) target women and men and occasionally the perceiver, there are good reasons to expect that gender role expectations will also affect group performance. Specifically, we argue that each of the three mechanisms *via* which gender role expectations reinforce themselves can shape group performance, such that group performance suffers to the extent to which gender role expectations inaccurately capture the division of expertise between men and women in gender-diverse work groups.

With regard to the allocation of jobs, tasks, and responsibilities, gender role expectations are likely to function as a heuristic that facilitates a task division among team members. However, as mentioned earlier, despite the general accuracy of gender role stereotypes regarding overall differences between women and men at the societal level (Jussim et al., 2015), gender role expectations will always carry a degree of inaccuracy in predicting the distribution of women and men’s knowledge and abilities in a specific gender-diverse work group for any given job, task, or responsibility. The more that gender role expectations inaccurately capture group members’ knowledge and abilities, the more likely it is that gender role expectations lead to a suboptimal task division. Because the performance of work groups tends to depend on the extent to which its members are allocated tasks that align with their expertise (Aime et al., 2014), we argue that the performance of a work group decreases the more that the allocation of jobs, tasks, and responsibilities is based on inaccurate gender role expectations.

Regarding the behavior of perceivers, the more inaccurate gender role expectations are, the more likely it is that perceivers in gender-diverse work groups will turn to the wrong persons for help, follow the wrong advice, and put their trust in those who cannot be trusted, which all inhibits performance. Furthermore, in being more influential, the less capable women and men in gender role-congruent positions are likely to yield an increase in errors and suboptimal decisions, also inhibiting performance. Indeed, given that groups tend to perform best when expertise is recognized (Bunderson, 2003; Joshi, 2014), we argue that the performance of a work group decreases the more the behavior of perceivers is based on inaccurate gender role expectations.

Finally, from the side of target women and men, the various ways in which women and men are pressured to display gender role-confirming behavior (i.e., by socialization, fear of backlash, or stereotype threat) diminishes the influence of women and men in gender role-incongruent positions on group processes and outcomes. If such women and men in reality are the most competent group members, we argue that their limited influence in the group is likely to harm the group’s performance. In line with this argument, a recent study showed that gender-diverse groups tended to perform worse to the extent that less-competent members were more influential (van Dijk et al., 2018). We thus argue that the performance of a work group decreases the more the behavior of target women and men is based on inaccurate gender role expectations.

In combination, we propose that gender role expectations harm group performance to the extent that gender role expectations inaccurately capture differences between male and female group members’ level of knowledge and abilities:

Proposition 4: The more inaccurately gender role expectations capture male and female group members’ knowledge and abilities, the more gender role expectations-based allocations of jobs, tasks, and responsibilities, behaviors of perceivers, and behaviors of target women and men inhibit group performance.

## Impression Formation Motivation as Key to Inhibit the Flywheel

Gender role expectations may at first glance appear a useful heuristic to assess one’s knowledge and abilities for a job, task or responsibility, yet they remain uninformed guesses at best. Meta-analytic studies on differences between women and men in most work-related knowledge and abilities in general tend to be small, heterogeneous, and converging (e.g., Eagly et al., 2003). More importantly, population differences say next to nothing about specific individuals.

Rather than relying on the flywheel of gender role expectations to form an impression of target persons, we therefore contend that individuals as well as work groups will benefit when group members use other means to discern knowledge and abilities. Based on the literature on how perceivers form impressions of target persons, we argue that group members’ impression formation *motivation* is crucial in changing perceivers’ reliance on gender role expectations in forming impressions of target persons.

Research on impression formation examines the process *via* which perceivers form an impression of a target. There are a number of slightly different models and theories on the process of impression formation (cf. Brewer, 1988; Fiske and Neuberg, 1990; Thagard and Kunda, 1996), but they all suggest that there are essentially two systems in a human brain that are responsible for forming an impression (Swencionis and Fiske, 2014). The first is the automatic or reflexive system that tends to form impressions automatically and often subconsciously by tapping into stereotypes in forming impressions of others. The second is the rational or reflective system that tends to form impressions based on deliberate attention to and the processing of individuating information.

Because the rational system consumes cognitive effort, perceivers tend to rely primarily on the automatic system in making inferences (Macrae et al., 1994). Accordingly, the general rule of impression formation is that impressions of others are mainly formed based on the automatic system, *unless* perceivers are sufficiently motivated to direct their attention to individuating information (Fiske and Neuberg, 1990; Nelson et al., 1996). The more that perceivers are motivated to form accurate impressions of others, the more they are willing to invest time and energy in looking beyond stereotype-based associations and pay attention to individuating information.

Gender role expectations are grounded in stereotypes. When perceivers rely on gender role expectations to make inferences of men and women, they thus tap into the automatic system. We therefore argue that the key to diverting work group members’ reliance on gender role expectations is to influence their impression formation motivation. The more that work group members are motivated to form accurate impressions of their fellow group members, the more they will rely on individuating information rather than gender role expectations in forming impressions of men and women.

Specifically, we expect that a motivation to form accurate impressions will inhibit the extent to which gender role expectations shape the allocation of jobs, tasks, and responsibilities, the behavior of perceivers, and the behavior of target men and women. In paying more attention to individuating information, the allocation of jobs, tasks, and responsibilities will be more based on who is the right person for the job in terms of actual knowledge and abilities, rather than inferred knowledge and abilities based on gender. In addition, perceivers will be more supportive of group members with actual knowledge and abilities and critical toward those with less knowledge and abilities, regardless of the gender role incongruity of such members (cf. Correll and Ridgeway, 2003; Wittenbaum and Bowman, 2005). We further expect that target women and men will feel less pressured to conform to gender role expectations and instead will feel free to display gender role-incongruent behavior when they experience the need to do so (e.g., when they are the most capable member of the group).

We thus argue that the motivation to form accurate impressions increases perceivers’ attention to individuating information and reduces their reliance on gender role expectations. The result is that (1) the allocation of group members to jobs, tasks, and responsibilities is more based on members’ knowledge and abilities, (2) the recognition of knowledge and abilities in work groups is improved, and (3) the most capable and experienced group members become more influential, which all positively affect group performance. We therefore propose:

Proposition 5: The more that perceivers are motivated to form accurate impressions of their work group members, the less gender role expectations will affect the allocation of jobs, tasks, and responsibilities, the behavior of perceivers, the behavior of target women and men, and will, in turn, enhance group performance.

## An Agenda for Future Research

In this conceptual analysis, we have argued that gender role expectations in work groups tend to behave like a flywheel. They automatically reinforce and maintain themselves *via* three mechanisms: the allocation of jobs, tasks, and responsibilities, the behavior of perceivers, and the behavior of target men and women. We have argued that this flywheel of gender role expectations will positively [negatively] affect group performance to the extent that gender role expectations accurately [inaccurately] capture differences in knowledge and abilities between men and women group members. In addition, we have argued that the performance of gender-diverse work groups benefits most when group members’ impression formation relies less on the flywheel of gender role expectations, and is instead grounded in individuating information. To make perceivers focus more on individuating information in forming impressions, we have argued that it is key to motivate them to focus on forming accurate impressions.

In combination, these propositions advance theory on gender role expectations and gender diversity in three ways. The first is in pointing out how gender role expectations in gender-diverse work groups tend to be self-reinforcing and operate like a flywheel. Second, we built theory regarding how gender role expectations shape the performance of diverse work groups. The third theoretical contribution pertains to how the motivation to form accurate impressions can reduce the influence of gender role expectations and enhance the performance of gender-diverse work groups. In the following, we present a research agenda for future research, which is structured along these three contributions.

## Advancing Research on the Flywheel of Gender Role Expectations

Years of research have shown how gender role expectations shape the allocation of jobs, tasks, and responsibilities, the behavior of perceivers, and the behavior of target men and women (e.g., Eagly and Karau, 2002; Correll and Ridgeway, 2003). These consequences of gender role expectations have been documented in a variety of domains (e.g., recruitment and selection, backlash, and stereotype threat). In clustering the findings of those studies on the consequences of gender role expectations into the three mechanisms of the flywheel of gender role expectations, we hope to have provided researchers with a useful categorization of the different consequences of gender role expectations.

However, we hope that future research will not only focus on these mechanisms as consequences of gender role expectations. The main reason why we introduced the analogy of a flywheel is because of the self-reinforcing nature of gender role expectations. We therefore put a premium on studies that move from a static way of studying the consequences of gender role expectations in isolation to approaches that enable an assessment of the dynamics of gender role expectations within work groups.

Such research requires designs that track the interaction of group members’ behavior in organizations over time. Researchers would need to measure gender role expectations longitudinally by using specifically designed indicators (e.g., specific behavioral expectations of group members for certain tasks, or indicators of automatic associations using instruments such as the Implicit Association Test; Greenwald et al., 1998) *via* repeated measures over time, and take stock of what happened in between that may account for changes in gender role expectations. For example, a male group leader may have been replaced by a female group leader, or group members may display more gender role-incongruent behavior. By complementing such findings with experiments in which the causality of the assumed underlying mechanisms is tested, researchers can assess the self-reinforcing nature of gender role expectations.

Although we presented and discussed each mechanism of the flywheel of gender role expectations independently, we expect that the three flywheel mechanisms also affect each other. First of all, the tendency to assign women and men to gender role-congruent jobs, tasks, and responsibilities prevents perceivers from being exposed to women and men in gender role-incongruent positions, and thus reinforces the gender role expectations of perceivers. Second, the gendered allocation of jobs, tasks, and responsibilities limits the extent to which individuals gain experience in gender role-incongruent positions. Third, the reciprocity in the interaction between perceivers and target men and women reinstates gender role expectations and their corresponding behaviors.

Preliminary evidence of such relationships among the mechanisms comes from a recent experimental study on task allocations, which showed that in gender-diverse groups, women, compared to men, more often tend to volunteer, are asked to volunteer, and accept requests to volunteer for low-status tasks (Babcock et al., 2017). Between gender-homogeneous groups, no such gender differences in the willingness to volunteer, the request to volunteer, or the acceptance of requests to volunteer existed. Findings also showed that gender role expectations, rather than individual preferences, were responsible for the gender differences in the behavior of the group members toward each other. Whereas we consider the three mechanisms to meaningfully distinguish between different ways in which gender role expectations maintain and reinforce themselves in work groups, we recommend researchers to also examine relationships among the three mechanisms.

## Advancing Research on the Group-Level Consequences of the Flywheel

Because almost all studies on the consequences of gender role expectations in organizational settings have focused on individual level behavior and outcomes (e.g., Hall et al., 2018), research on how gender role expectations shape group-level behaviors and outcomes is still in its infancy. However, we contend that such research is important, given that the interest of many practitioners in diversity tends to focus primarily on how diversity shapes organizational performance (cf. Catalyst, 2004; Eagly, 2016). Two related studies show what research on the relationship between gender role expectations and work group behavior and performance can look like – and how it can advance our knowledge about the consequences of gender role expectations in organizations.

Chatman et al. (2008) showed that the behavior of gender-diverse groups depends on the gender distribution in relation with the nature of the task. Group members who were the only representative of their gender were assumed to be the most competent group member on gender role-congruent tasks (cf. Kanter, 1977; van Knippenberg et al., 2004), and were therefore more often deferred to (cf. Sekaquaptewa and Thompson, 2003). In a similar experiment, van Dijk et al. (2018) showed that group members on gender role-congruent tasks in gender-diverse groups were more influential (measured by speaking time) compared to group members on gender role-incongruent tasks during discussions. In the work groups where gender role-expectations did not match the actual competence of the group members (e.g., the male group member was lower in math ability than the female group members), group members followed the wrong lead (e.g., not using the correct math resolutions offered by competent women, but following men’s suggestions in the group), and group performance decreased.

The findings of Chatman et al. (2008) and van Dijk et al. (2018) provide preliminary evidence that gender role expectations shape interactions and performance at the group level. Moreover, they challenge the long-standing proposition in diversity research that diverse groups should be able to make better decisions compared to homogeneous groups when they discuss and share the richness and variety in knowledge, information, and perspectives present in their group (van Knippenberg et al., 2004): in deciding which information to ignore and whose advice to heed, group members tend to rely on biases and heuristics such as gender role expectations rather than being able to objectively assess the value and merit of each member’s contribution.

Controlled experiments can build on the studies by Chatman et al. (2008) and van Dijk et al. (2018) to further establish the causal mechanisms of gender role expectations in the functioning and performance of groups. The paucity of research in this area provides numerous opportunities for future research. However, given their importance for team performance, we consider it especially important for future research in this area to further examine the processes and conditions that cause group members to weigh contributions based on gender role expectations – and what may make them forsake doing that.

Furthermore, field research in which work groups in organizations are followed over time would be necessary to examine the extent to which laboratory studies translate to organizational contexts. For instance, work group meetings could be observed to capture verbal and non-verbal expressions of gender role expectations among perceivers as well as target men and women. In relating such behaviors to meeting outcomes and work group performance over time, researchers can assess how gender role expectations may shape work group performance in organizational work groups.

## Advancing Research on Ways to Motivate Perceivers to Form Accurate Impressions

We have argued that motivating perceivers to form accurate impressions will reduce their reliance on gender role expectations and inhibit its flywheel effect. Theory suggests that perceivers’ impression formation motivation depends on (1) what the perceiver wants, (2) who controls what the perceiver wants, and (3) what the criteria are for attaining the desired outcome (Fiske and Neuberg, 1990; van Dijk et al., 2017). For example, if a group member desires to be promoted and her or his manager is in charge of making that call, then it is likely that the group member will follow the criteria that the manager has set for promotion. If those criteria include work group elements (e.g., group performance, getting along well with the other group members), then it is more likely that the group member will invest in getting to know the other group members compared to when the criteria only focus on the individual performance of the group member (cf. Overbeck and Park, 2001). Because there is hardly any research in organizations that has looked at how perceivers’ motivation to form accurate impressions and reliance on individuating information can be enhanced, we argue that these theoretical guidelines provide a good start for future research.

However, given that there is a large variety in organizational contexts that can relate to differences in what perceivers want (e.g., public versus private sector), who controls what the perceiver wants (e.g., manager, other team members, client), and which contextual factors are known to shape perceivers’ impression formation (e.g., task complexity, level of interaction, accountability), many studies will be needed to gather conclusive empirical evidence regarding the criteria that stimulate the motivation to form accurate impressions across task contexts. We therefore recommend researchers to adopt a collaborative approach in studying how perceivers’ motivation to form accurate impressions can be enhanced in gender-diverse work groups. An inspirational example of this kind of research is a comparative study by Lai et al. (2016) which reports on a research contest in which research teams were invited to test interventions to reduce implicit racial bias (as measured by the IAT). Extending such a research design to examine the formation of accurate impressions as a function of manipulations of impression formation motivation would provide rich data on possible criteria that may drive the formation of accurate impressions in work groups and inhibit the flywheel of gender role expectations.

Furthermore, research on diversity in organizations suggests that the performance of (gender-)diverse work groups is facilitated by fostering a diversity climate (e.g., Shore et al., 2011; Nishii, 2013), which refers to “employees’ perceptions about the extent to which their organization values diversity as evident in the organization’s formal structure, informal values, and social integration of underrepresented employees” (Dwertmann et al., 2016, p. 1137). The exact reasons why diversity climates enhance the performance of diverse work groups are still subject of debate and study, but it could very well be that diversity climates in gender-diverse work groups enhance perceivers’ motivation to form accurate impressions.

Specifically, Dwertmann et al. (2016) suggested that a diversity climate consists of two components. The *fairness and discrimination* component is defined as “shared perceptions about the extent to which the organization and/or workgroup successfully promotes fairness and the elimination of discrimination through the fair implementation of personnel practices, the adoption of diversity-specific practices aimed at improving employment outcomes for underrepresented employees, and/or strong norms for fair interpersonal treatment” (p. 1151). The *synergy* component of a diversity climate refers to “the extent to which employees jointly perceive their organization and/or workgroup to promote the expression of, listening to, active valuing of, and integration of diverse perspectives for the purpose of enhancing collective learning and performance” (p. 1151). Although each component thus has a different focus and purpose, they both require the organization to establish strong norms that they actively promote and reinforce. To institutionalize such strong norms, criteria involving adherence to such norms and accountability are essential – factors that have been suggested to enhance perceivers’ motivation to form accurate impressions (Tetlock, 1983; Fiske and Neuberg, 1990).

Interestingly, the fairness and discrimination component is likely to inhibit the extent to which gender role expectations shape the allocation of jobs, tasks, and responsibilities, whereas the synergy component is likely to inhibit the extent to which gender role expectations shape the behaviors of perceivers and of target men and women. As such, the establishment of a diversity climate may provide an integral solution to motivate perceivers to form accurate impressions, inhibit the flywheel of gender role expectations, and enhance the performance of (gender-)diverse work groups. We therefore recommend that researchers tap into this potentially fruitful avenue for future research.

## Conclusion

In using a flywheel as an analogy to illustrate the self-reinforcing nature of gender role expectations in gender-diverse work groups, we hope to create awareness about the pervasiveness of gender role expectations. Moreover, in pointing out that individuals as well as work groups can suffer from gender role expectations, we hope to establish a sense of urgency about the importance of addressing ways to inhibit the flywheel of gender role expectations. We call for researchers as well as practitioners to work together in assessing which interventions are effective in helping members of gender-diverse work groups to rely less on the flywheel of gender role expectations and motivate them to form accurate impressions instead.

## Author Contributions

HD conceptualized the flywheel analogy. HD and ME co-authored the manuscript.

### Conflict of Interest Statement

The authors declare that the research was conducted in the absence of any commercial or financial relationships that could be construed as a potential conflict of interest.
